# Lewy‐related pathology in the Tampere Sudden Death Study

**DOI:** 10.1002/alz.094657

**Published:** 2025-01-09

**Authors:** Eloise H Kok, Anders Paetau, Mika Martiskainen, Leo‐Pekka Lyytikäinen, Terho Lehtimäki, Pekka Karhunen, Liisa Myllykangas

**Affiliations:** ^1^ University of Helsinki, Helsinki Finland; ^2^ Tampere University, Tampere Finland; ^3^ University of Helsinki, Helsinki, Uusimaa Finland; ^4^ HUS Diagnostic Center, Helsinki, Uusimaa Finland; ^5^ Finnish Institute for Health and Welfare, Helsinki Finland; ^6^ Fimlab Laboratories, Tampere Finland

## Abstract

**Background:**

When treatments against neurodegenerative diseases eventuate, the most successful time point to administer therapies will be the early stages, most likely before symptoms begin to show. Previous studies have found alpha‐synuclein pathology (Lewy‐related pathology; LRP) in asymptomatic individuals aged 62 years and above, with ranges from 8 to 17%.

**Method:**

We performed immunohistochemical staining with the 5G4 antibody against alpha‐synuclein on brain tissue samples from the Tampere Sudden Death Study – a collection of forensic autopsies on individuals that lived outside hospital institutions in Tampere and the surrounding regions, Finland. Representative paraffin‐embedded tissue regions of brainstem (substantia nigra/pons) were available from 562 cases, ranging in age from 16 to 95 years, with a majority of men (74% vs 145 women). Hippocampal regions were available in 476 cases (87% of those with brainstem available). (Kok et al. 2024)

**Result:**

Alpha‐synuclein pathology was seen in 46 individuals with varying severity. Of the 46 cases positive for alpha‐synuclein pathology, the youngest (42y female) had clinical records of parkinsonism and neuropathologically determined as multiple system atrophy (MSA). An additional 3 cases also showed MSA‐type glial cytoplasmic inclusions, without clinical history. 42 subjects showed Lewy‐related pathology (LRP) in the brainstem. The youngest case with LRP was 54 years with no reported neurodegenerative issues (see Figure). One positive brainstem case was missing the hippocampal region, but otherwise 25 (61%) also had LRP in hippocampal regions. Surprisingly, 9% of those aged 50 years or older had LRP, with a clear preference for males (31 vs 11 females) and increasing incidence with age.

**Conclusion:**

This is the first investigation of a non‐hospitalised population across the adult life span showing the prevalence of LRP in individuals aged as young as their 50’s, suggesting LRP starts much earlier than previously thought and is more common than expected in middle age. The present study has the strength of a large number of younger cases, although may over represent drug abuse and psychiatric issue patients, as well as the obviously higher number of males.

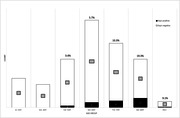

**Figure 1. Lewy‐related pathology prevalence according to age in the Tampere Sudden Death Study cohort. Asyn = alpha‐synuclein**.

